# Be original – You were born one

**DOI:** 10.4103/0972-0707.48833

**Published:** 2008

**Authors:** Velayutham Gopikrishna

**Affiliations:** Journal of Conservative Dentistry, Department of Conservative Dentistry, Meenakshi Ammal Dental College, Alapakkam Main Road, Maduravoyal, Chennai–600 095, Tamilnadu, India. Email:editor@jcd.org.in

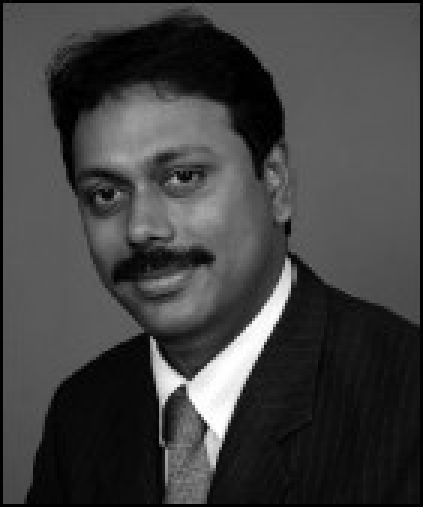



All of us want to be original, because nobody likes to be a copy! Deep inside, each one of us wants to be an A. R. Rahman, a Mother Teresa, an Abdul Kalam or even a Mahatma. However, the truth is that we get lost in and amongst the crowd of reality.

Every one starts out in his/her professional life with all the zeal and enterprise, but loses steam as the journey unwinds. It is so easy to give up, so easy to blame it on someone else, so easy to blame the system, and so easy to remain mediocre. Herein lies the biggest challenge for any professional – how to escape “excusitis” and “mediocrity”? The answer to this lies in having “self-belief” and in “remaining original”.

## SELF BELIEF

As a teacher, I find that the difference between a performing and underperforming student is neither lack of knowledge nor lack of skill. It is always lack of “self-belief”. Hence, even if no one believes in you, you have got to believe in yourself. Even if no one believes you can, you have to believe you can. All in all, how the world sees you will make only a small difference to you, but how you see yourself will make all the difference!

## REMAINING ORIGINAL

Now even if one has self-belief, how can one be original in endodontics?

One of my students remarked, “It is sometimes so boring and monotonous doing root canal after root canal. It is so mechanical!” However, the fact remains that boredom and monotony are in the minds of men and not in life itself. When a tulip blooms, it is not a repetition of any tulip that has preceded it. It is original, unique, and incomparable. In the same spirit each one of us is born unique and original. Similarly, every clinical case we encounter brings along with it, its own challenges. My teacher once remarked, “A molar never walks into your clinic! It is a human being going through one of his/her most agonizing moments in life, who needs your professional expertise.”

Hence, you remain original by being sincere and passionate in the work you do. You remain original by doing research on newer uncharted territories rather than taking a “key article” and repeating the same study. You remain original by considering your dissertation as a small, but important step, in improving our understanding of the science of Conservative Dentistry and Endodontics. You remain original by setting your own standards rather than matching them to the set standards.

In any profession there are a handful of originals and a universe of copies! This is what sets apart a Grossman, a Gutmann, a Weine or a Schilder from others.

So ask yourself this question,

“Are you destined to be a copy or are you going to change your destiny to remain original?”

